# Effects of Ultramicronized *N*-Palmitoylethanolamine Supplementation on Tramadol and Oxycodone Analgesia and Tolerance Prevention

**DOI:** 10.3390/pharmaceutics14020403

**Published:** 2022-02-11

**Authors:** Laura Micheli, Elena Lucarini, Alessandra Toti, Valentina Ferrara, Clara Ciampi, Carmen Parisio, Gianluca Bartolucci, Lorenzo Di Cesare Mannelli, Carla Ghelardini

**Affiliations:** 1 Department of Neuroscience, Psychology, Drug Research and Child Health-Neurofarba-Pharmacology and Toxicology Section, University of Florence, 50139 Florence, Italy; laura.micheli@unifi.it (L.M.); elena.lucarini@unifi.it (E.L.); alessandra.toti@unifi.it (A.T.); valentina.ferrara@unifi.it (V.F.); clara.ciampi@unifi.it (C.C.); carmen.parisio@unifi.it (C.P.); carla.ghelardini@unifi.it (C.G.); 2 Department of Neuroscience, Psychology, Drug Research and Child Health-Neurofarba-Pharmaceutical and Nutraceutical Sciences Section, University of Florence, 50019 Florence, Italy; gianluca.bartolucci@unifi.it

**Keywords:** pain therapy, opioid, tolerance, astrocyte, PEA, tramadol, oxycodone

## Abstract

Chronic pain management requires increasing doses of opioids, the milestone of painkillers, which may result in the onset of tolerance with exacerbated side effects. Maintaining stable analgesia with low doses of opioids is thus imperative. *N*-palmitoylethanolamine (PEA) is an endogenous lipid compound endowed with pain-relieving as well as anti-inflammatory properties. The ultramicronized formulation of PEA was recently demonstrated to be able to modulate morphine’s effects, delaying tolerance and improving efficacy. To evaluate the possible application to other opioids, in this study, we analysed the capacity of ultramicronized PEA to regulate analgesia and tolerance induced by oxycodone and tramadol. Pre-emptive and continuative treatment with ultramicronized PEA (30 mg kg^−1^, daily, per os) delayed the onset of opioid tolerance and enhanced opioid analgesia when it was acutely administered in association with tramadol (20 mg kg^−1^, daily, subcutaneously) or oxycodone (0.5 mg kg^−1^, daily, subcutaneously). Moreover, PEA exerted antinociceptive effects on tolerant rats, suggesting the use of PEA together with opioids for stable, long-lasting analgesia. To that purpose, the oxycodone dose needed to be increased from 0.3 mg kg^−1^ (day 1) up to 1 mg kg^−1^ (day 31) in the oxycodone + vehicle group; the tramadol dose was progressively enhanced from 15 mg kg^−1^ to 50 mg kg^−1^ in 31 days in the tramadol + vehicle group. Acute oral co-treatment with PEA (120 mg kg^−1^) achieved the same analgesia without increasing the dose of both opioids. The behavioural effects of PEA on opioid chronic treatment paralleled a decrease in astrocyte activation in the dorsal horn of the spinal cord (a marker of the development of opioid tolerance) and with a modulation of mRNA expression of IL-6 and serpin-A3. In conclusion, pre- and co-administration of ultramicronized PEA delayed the development of tramadol tolerance, potentiating either oxycodone or tramadol analgesia and allowing a long-lasting analgesic effect with a low opioid dose regimen. The use of PEA is suggested for clinical purposes to support the opioid-based management of persistent pain.

## 1. Introduction

Pain is considered to be a global public health problem, since the burden of chronic and acute pain is remarkable and constantly increasing. Chronic pain commands attention above all, since it is usually difficult to treat, and there is currently an ongoing debate as to whether persistent pain has to be considered as a disease in its own right. Pain control has been postulated as a ‘core ethical duty in medicine’ [1], and the International Association for the Study of Pain (IASP) took a stand on the question of ethics and pain, by adopting the Declaration of Montreal, which states that access to pain management is a fundamental human right [2]. To this end, the World Health Organization (WHO) recognized the usefulness of opioid analgesics [3], and a consensus among several international bodies such as the United Nations Economic and Social Council and the World Health Assembly was gained [3].

However, the overprescription and overmarketing of opioids in areas such as North America, Australia and Europe has led to the ‘opioid crisis’ [4]. Indeed, although opioids are very effective painkillers, their collateral effects need a careful approach in chronic pain states [5]. To avoid tolerance, increasing doses of opioids are required to maintain a sufficient analgesic effect. The dose escalation exacerbates the severity of opioids’ side effects such as dizziness, sedation, nausea, constipation, vomiting, cognitive impairments, physical dependence, dry mouth and respiratory depression [6]. For these reasons, limiting the development of opioid tolerance while keeping the lowest effective dosage could be a valid strategy to reduce opioids’ side effects during a course of chronic therapy.

Palmitoylethanolamide (PEA) is an endogenous lipid mediator belonging to the *N*-acylethanolamine (NAE) family, which plays a local autacoid role in controlling inflammation and in analgesic phenomena [7]. Provided that bioavailable formulations are used, PEA administration counteracts neuronal alterations, reduces morphine tolerance [8] and potentiates morphine analgesia [9]. Indeed, in a pre-clinical study, repeated PEA treatment was able to delay the onset of morphine tolerance by acting on glial cells that are a keystone of the tolerance phenomenon [10,11]. The plasticity of astrocytes and microglia is also a characteristic of neuropathic pain states. PEA, as a modulator of the glial response [12], is active against different models of neuropathy [13], as well as naturally occurring chronic pain in human and veterinary patients, as recently reviewed in [14]. Moreover, PEA protects against neurotoxicity and neuroinflammation [15,16,17,18,19] and downmodulates mast cell degranulation [7,20].

All these properties lay the groundwork for the use of PEA during opioid-based therapies. Clinically speaking, the possibility of using PEA as a powerful enhancer and modulator of opioid analgesia and tolerance, respectively, would lead to a revolution in chronic pain management. To this purpose, we evaluated the use of ultramicronized PEA [21] as a modulator of oxycodone and tramadol tolerance. Moreover, we set up a protocol for PEA supplementation during repeated tramadol and oxycodone treatments with the aim of maintaining, over days, a stable antinociception with the lowest opioid regimen. At the end, an evaluation of glial cells was performed and the effect of PEA treatment on histamine plasma levels was examined.

## 2. Materials and Methods

### 2.1. Animals

Male Sprague–Dawley rats (Envigo, Varese, Italy), weighting approximately 250–300 g, were used for all the experiments described below. Rats were housed in CeSAL (Centro Stabulazione Animali da Laboratorio, University of Florence) and used at least 1 week after their arrival. Four rats were housed per cage (size 26 × 41 cm) and kept at 23 ± 1 °C with a 12 h light/dark cycle, with light at 7 a.m. Animals were fed ad libitum with a standard laboratory diet and tap water.

### 2.2. Study Design

**Figure 1:**
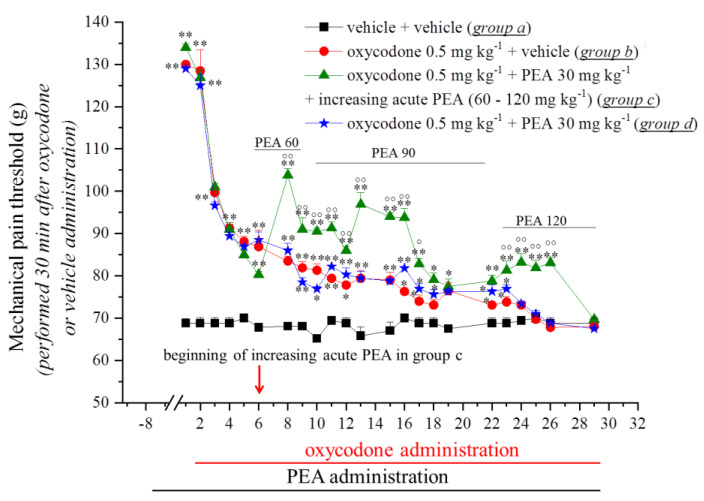
Effect of repeated administration of PEA on the onset of oxycodone tolerance and analgesia. Pre-emptive PEA (30 mg kg^−1^; Groups c and d) or the vehicle (Groups a and b) was orally administered daily (in the evening) for the duration of the experiment, starting on Day −8. On Day 1, daily, acute oxycodone treatment (0.5 mg kg^−1^ s.c.) started. From Day 6, Group c received an additional acute daily PEA treatment (60–120 mg kg^−1^) in co-administration with the opioid. The pain threshold measurements (paw pressure test) were conducted in the morning, 16 h after pre-emptive PEA administration and 30 min after oxycodone injection. The results are expressed as the mean ± S.E.M. of values from 12 rats analysed in two different experimental sets. Statistical analysis was one-way ANOVA followed by Bonferroni’s post hoc comparison. * *p <* 0.05 and ** *p <* 0.01 vs. vehicle + vehicle; ° *p <* 0.05 and °° *p <* 0.01 vs. oxycodone + vehicle.

**Figure 2:**
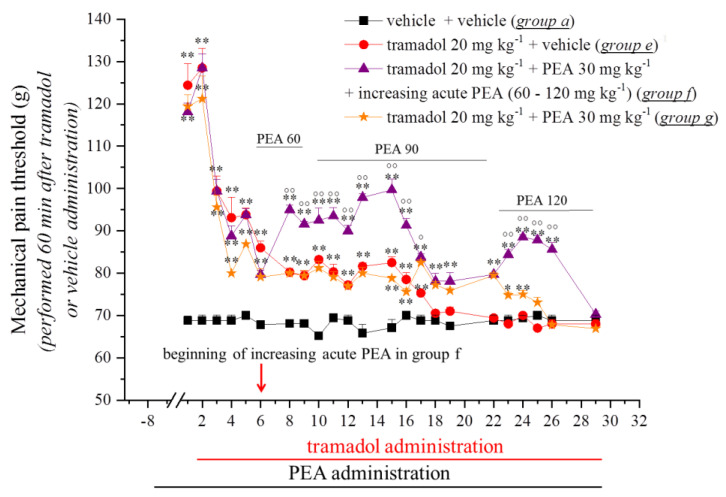
Effect of repeated administration of PEA on the onset of tramadol tolerance and analgesia. Pre-emptive PEA (30 mg kg^−1^; Groups f and g) or the vehicle (Groups a and e) was orally administered daily (in the evening) for the duration of the experiment, starting on Day −8. On Day 1, daily, acute tramadol treatment (20 mg kg^−1^ s.c.) started. From Day 6, Group f received an additional acute daily PEA treatment (60–120 mg kg^−1^) in co-administration with the opioid. The pain threshold measurements (paw pressure test) were conducted in the morning, 16 h after pre-emptive PEA administration and 60 min after tramadol injection. The results are expressed as the mean ± S.E.M. of values from 12 rats analysed in two different experimental sets. Statistical analysis was one-way ANOVA followed by Bonferroni’s post hoc comparison. * *p <* 0.05 and ** *p <* 0.01 vs. vehicle + vehicle; ° *p <* 0.05 and °° *p <* 0.01 vs. tramadol + vehicle.

**Figure 3:**
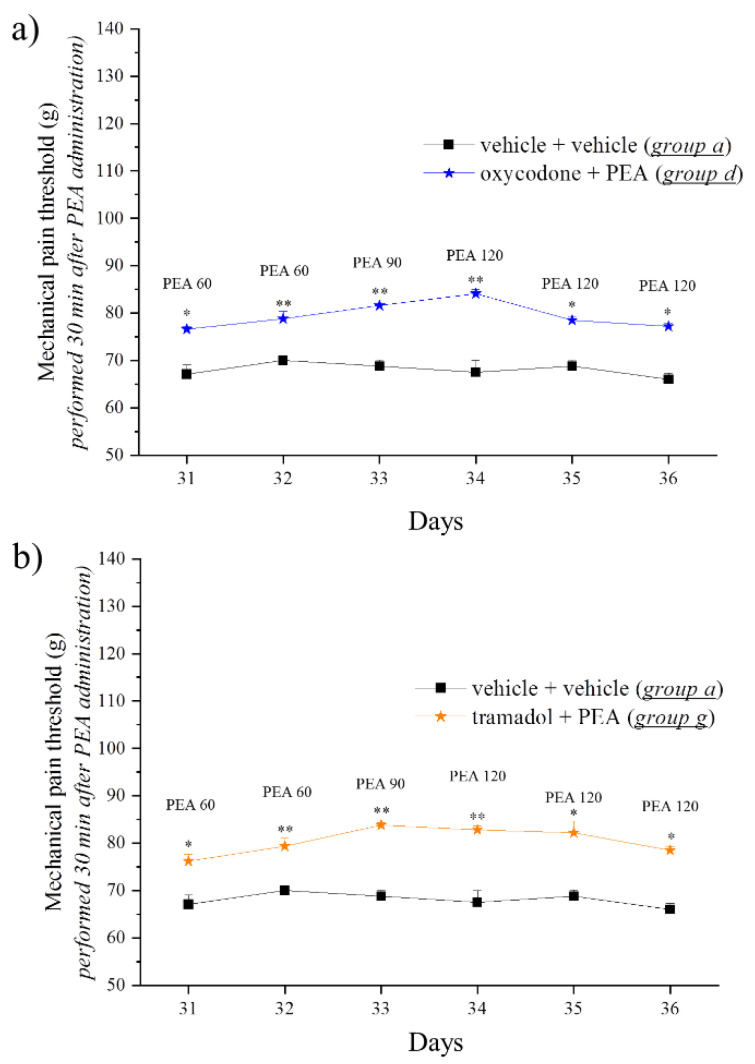
Effect of acute administration of PEA on opioid-tolerant animals. Tolerance was induced by treating rats with (**a**) oxycodone (0.5 mg kg^−1^ s.c.; Group d) or (**b**) tramadol (20 mg kg^−1^ s.c.; Group g) from Day 1 to Day 29. Pre-emptive PEA (30 mg kg^−1^) was orally administered daily (in the evening) for the duration of the experiment (Days −8 to 29) in Groups d and g. On Day 31, when tolerance to oxycodone or tramadol was well established, the pain threshold was measured in the morning, before and 30 min after acute treatment with PEA (60–120 mg kg^−1^, Days 31–36), by the paw pressure test. The results are expressed as the mean ± S.E.M. of values from 12 rats analysed in two different experimental sets. Statistical analysis was one-way ANOVA followed by Bonferroni’s post hoc comparison. * *p <* 0.05 and ** *p <* 0.01 vs. vehicle + vehicle, 30 min.

In the first behavioural experiments (Figure 1, Figure 2 and Figure 3), ultramicronized PEA (Epitech Group, Padova, Italy, 30 mg kg^−1^ suspended in 1% carboxymethylcellulose (CMC); Groups c, d, f and g) or the vehicle (Groups a, b and e) were administered p.o. daily (in the evening) from Day −8 till the end of the behavioural experiments. Starting on Day 1, up to the development of tolerance, acute treatments with oxycodone (Molteni, Florence, Italy; 0.5 mg kg^−1^ dissolved in 0.9% NaCl s.c.; Groups b, c and d) or tramadol (Grunenthal, Milan, Italy; 20 mg kg^−1^ dissolved in 0.9% NaCl s.c.; Groups e, f and g) were administered in the morning, 16 h after PEA. The pain threshold measurements (paw pressure test) were made before (0 min) and 30 or 60 min after oxycodone or tramadol injection, respectively. From Day 6 till the end of the experiments, Groups c and f received increasing acute doses of PEA (60–120 mg kg^−1^) together with the opioids to preserve a significant increase in the pain threshold. Tolerance started the on first day of the complete loss of the opioids’ antinociceptive effect (Day 23 for Groups b and c, Day 27 for Group d, Day 17 for Group e, Day 26 for Group f, Day 24 for Group g). 

**Scheme 1:**
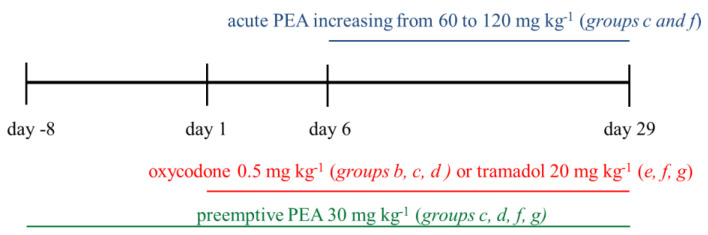
Representative image of the first experimental treatment protocol.

A schematic summary of the first experimental treatment protocol is shown in Scheme 1.

Group a: vehicle + vehicle (from Day −8 to Day 29);
Group b: 0.5 mg kg^−1^ oxycodone (from Day 1 to Day 29) + vehicle (from Day −8 to Day 29);
Group c: pre-emptive PEA (30 mg kg^−1^ from Day −8 to Day 29) + 0.5 mg kg^−1^ oxycodone (from Day 1 to Day 29) + increasing acute PEA (60–120 mg kg^−1^ from Day 6 to Day 29);
Group d: pre-emptive PEA (30 mg kg^−1^ from Day −8 to Day 29) + 0.5 mg kg^−1^ oxycodone (from Day 1 to Day 29);
Group e: 20 mg kg^−1^ tramadol (from Day 1 to Day 29) + vehicle (from Day −8 to Day 29);
Group f: pre-emptive PEA (30 mg kg^−1^ from Day −8 to Day 29) + 20 mg kg^−1^ tramadol (from Day 1 to Day 29) + increasing acute PEA (60–120 mg kg^−1^ from Day 6 to Day 29);
Group g: pre-emptive PEA (30 mg kg^−1^ from Day −8 to Day 29) + 20 mg kg^−1^ tramadol (from Day 1 to Day 29).

Moreover, the antinociceptive effect of acute PEA (60–120 mg kg^−1^) was evaluated in oxycodone and tramadol tolerant animals in Groups d and g from Day 31 to Day 36 (Figure 3a,b).

In the second phase, experiments (Figures 5–9) were performed on different groups of animals treated, respectively, with the vehicle (Group a) or PEA (30 mg kg^−1^; Groups b, c, d and e) p.o. daily (in the evening) from Day −8 to the end of the experiments. To maintain a significant and stable pain threshold (90 ± 10 g) vs. the control group, daily, beginning on Day 1, increasing doses of oxycodone (0.3–1 mg kg^−1^) or tramadol (15–50 mg kg^−1^) were injected s.c. into Groups b or d (Figures 5 and 6, respectively). Different combinations of oxycodone (0.3 mg kg^−1^, s.c.) and PEA (30–90 mg kg^−1^, p.o.), or tramadol (15 mg kg^−1^) and PEA (30–90 mg kg^−1^, p.o.) were administered to Groups c and e, respectively. The control group was pre-treated and acutely treated with the vehicle (Group a). On Days 1–31, pain measurements were performed in the morning, 30 min after opioid and/or acute PEA administration.

**Scheme 2:**
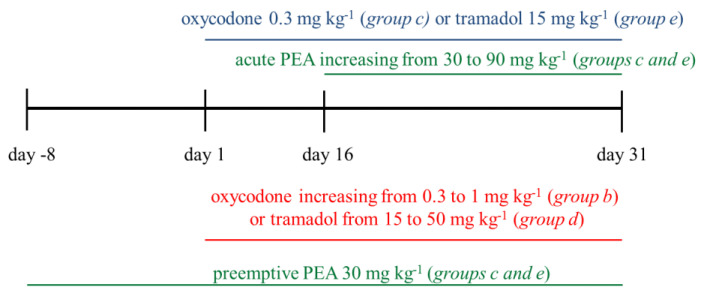
Representative image of the second experimental treatment protocol.

A schematic summary of the second experimental treatment protocol is shown in Scheme 2.

Group a: vehicle + vehicle (from Day −8 to Day 31);
Group b: increasing oxycodone (0.3–1 mg kg^−1^ from Day 1 to Day 31) + vehicle (from Day −8 to Day 31);
Group c: pre-emptive PEA (30 mg kg^−1^ from Day −8 to Day 31) + 0.3 mg kg^−1^ oxycodone (from Day 1 to Day 31) + increasing acute PEA (30–90 mg kg^−1^ from Day 16 to Day 31);
Group d: increasing tramadol (15–50 mg kg^−1^ from Day 1 to Day 31) + vehicle (from Day −8 to Day 31);
Group e: pre-emptive PEA (30 mg kg^−1^ from Day −8 to Day 31) + 15 mg kg^−1^ tramadol (from Day 1 to Day 31) + increasing acute PEA (30–90 mg kg^−1^ from Day 16 to Day 31).

### 2.3. Paw Pressure Test

An analgesimeter (Ugo Basile, Varese, Italy) was used to measure the nociceptive pain threshold of rats according to [22]. We applied a constantly increasing pressure on the dorsal surface of the hind paw using a blunt conical probe by a mechanical device. The pressure was increased until vocalization or a withdrawal reflex occurred while rats were lightly restrained. Vocalization or withdrawal reflex thresholds were expressed in grams. Rats scoring below 40 g or over 75 g during the test before drug administration were rejected (25%). A cut-off of 200 g was adopted. Starting on Day 1 and until the end of the experiments, the paw pressure test was performed twice daily, before and after the injection of tramadol or oxycodone.

### 2.4. Tissue Collection

At the end of the behavioural experiments, animals were killed by decapitation. The lumbar spinal cord was collected, frozen with liquid nitrogen or fixed by immersion in 4% formalin for PCR or immunohistochemical analysis, respectively.

### 2.5. Immunohistochemistry of the Spinal Cord

Formalin-fixed cryostat sections of the lumbar spinal cord (5 μm) were incubated for 1 h in a blocking solution (Bio-Optica; Milan, Italy) at room temperature; thereafter, sections were incubated for 24 h at 4 °C in PBST containing primary antisera and 5% normal donkey serum. The primary antibody was directed against glial fibrillary acidic protein (GFAP; rabbit antiserum, 1:500; Dako, Santa Clara, CA, USA; [23]) for astrocyte staining. After rinsing in PBST, sections were incubated in donkey anti-rabbit IgG secondary antibody labelled with Alexa Fluor 568 (1:1000, Invitrogen, Waltham, MA, USA) at room temperature for 1 h.

Negative control sections (no exposure to the primary antisera) were analysed concurrently with the other sections.

In each animal, a single optical density value for the dorsal horns was obtained by averaging the two sides. The data thus obtained were compared with the homologous average values from the vehicle-treated animals.

### 2.6. Quantitative Analyses of GFAP Immunohistochemistry

A motorized Leica DM6000B microscope (Leica Microsystems, Wetzlar, Germany) equipped with a DFC350FX camera (Leica Microsystems, Wetzlar, Germany) was used to acquire the images. Quantification of the GFAP signal in immunostained sections was performed by FIJI software using automatic thresholding images with the aid of the ‘Moments’ algorithm, which delivered the most consistent pattern recognition across all acquired images.

### 2.7. Real-Time Polymerase Chain Reaction (RT-PCR)

Total RNA was isolated from the rats’ spinal cord using TRI Reagent (Merck, Milan, Italy). One microgram of RNA was retrotranscribed using the PrimeScript^TM^ RT reagent Kit with a gDNA eraser (Takara Bio, San Jose, CA, USA). RT-PCR was performed using SsoAdvanced Universal SYBR^®^ Green Supermix (Bio-Rad, Hercules, CA, USA) following the thermal profile suggested by the kit. The following primers were used: rSerpin-A3: forward, 5′-CTTTCTGCAGTATGTGGGAATCACTTGG-3′; reverse, 5′-GGCTGCATTGCTCTAAGTAGGAGTGC-3′; rEAAT1: forward, 5′-CAGTCATCGTCGGCCTCCTCATTC-3′; reverse, 5′-CTGGTGATGCGTTTGTCCACACCATTG-3′ (Integrated DNA Technologies, Coralville, IA, USA). Validated primers for rIL-6, rGAPDH and rβ-actin were purchased from Bio-Rad (qRnoCID0053166, qRnoCID0057018, qRnoCID0056984). The differential expression of the transcripts was normalized to the housekeeping gene GAPDH and β-actin.

### 2.8. Histamine Dosage by HPLC-MS/MS

The concentration of histamine in rat plasma was determined by the isotopic dilution (ID) two-dimensional (2D) HPLC-MS/MS method with a triple quadrupole mass spectrometer (QqQ). The solvents used for the HPLC mobile phase were:

Solvent A: 10% CH_3_CN 5 mM HCOOH 15 mM HCOONH_4_;
Solvent B: 90% CH_3_CN 15 mM HCOOH 5 mM HCOONH_4_;
Solvent C (used for sample loading): 90% CH_3_CN 17.5 mM HCOOH 2.5 mM HCOONH_4_.

The columns used for the histamine 2D-HPLC assay were the SeQuant^®^ ZIC-HILIC 50 × 2.1 mm, 3.5 µm, 100 Å (analytical column) and the SeQuant^®^ ZIC-HILIC Guard 20 × 2.1 mm (loading column).

The total analysis time was 25 min per sample; the samples were prepared by simple dilution 1:10 (*v*/*v*) in Solvent C.

### 2.9. Statistical Analysis

Observers blinded to the treatments carried out the tests. The results were expressed as means ± S.E.M., and analysis of variance was performed by a one-way ANOVA test. As a post hoc comparison, Bonferroni’s significant difference procedure was used; *p*-values less than 0.05 were considered significant. Data were analysed using Origin 9.1 software.

## 3. Results

### 3.1. PEA Modulates the Onset of Tramadol and Oxycodone Antinociception and Tolerance

In the first experiment, we decided to explore the capacity of PEA to modulate the onset of oxycodone and tramadol tolerance and to intervene in their analgesic effect. Rats were treated with PEA (30 mg kg^−1^, p.o.) daily (in the evening; Days −8–29). On Day 1 (16 h after the last PEA treatment), oxycodone (0.5 mg kg^−1^) or tramadol (20 mg kg^−1^ s.c.) was acutely injected. The animal’s pain threshold was measured twice daily using a noxious mechanical stimulus (the paw pressure test) before (0 min; data not shown) and after opioid injection. The values reported in Figure 1 and Figure 2 relate to the measurements taken 30 or 60 min later after oxycodone or tramadol administration, respectively. The pre-treatment with PEA did not improve the analgesic effect of the opioids that elevated the animal’s pain threshold up to 130 g in comparison with 70 g in the control group (*p <* 0.01) (Figure 1 and Figure 2). During the following days, the analgesic efficacy of oxycodone and tramadol progressively decreased. On Day 18, tramadol + vehicle-treated animals (Group e) completely lacked an analgesic response, whereas rats pre-treated with PEA (Group g) showed a delay in the onset of tolerance until Day 24 (*p <* 0.01) (Figure 2). On the contrary, PEA did not alter the onset of tolerance in oxycodone + PEA-treated animals (Group d), which appeared on Day 23 as well as in the oxycodone + vehicle group (Group b) (Figure 1). Based on previous evidence in which the acute administration of PEA, together with morphine, was able to potentiate the analgesic efficacy of the opioid [9], we performed an acute treatment with increasing doses of PEA (60–120 mg kg^−1^) (together with oxycodone and tramadol and in addition to the pre-emptive PEA treatment) in Groups c and f starting on Day 6 until Day 29. As shown in Figure 1 and Figure 2, the co-administration of PEA with oxycodone (Group c) or tramadol (Group f) potentiated the analgesic efficacy of the opioids, significantly increasing the pain threshold of the animals in comparison to Groups b (*p <* 0.05 and *p <* 0.01) (oxycodone + vehicle) and e (*p <* 0.05 and *p <* 0.01) (tramadol + vehicle). Moreover, a delay in the development of tolerance in these groups was also recorded.

Since it has been reported that PEA is able to counteract neuropathic pain without modifying the normal pain threshold [24], and since the conditions of neuropathy share several similarities with opioid tolerance such as glial cell alterations [11,25,26], we then tested the capacity of PEA to determine analgesia in tolerant animals. On Day 31, tolerant animals from Group d (oxycodone + PEA; Figure 3a) and from Group g (tramadol + PEA; Figure 3b) were acutely injected with PEA 60 mg kg^−1^ and the pain threshold was measured before (0 min) and 30 min after administration using the paw pressure test. PEA administration evoked analgesia in both groups, significantly increasing the weight tolerated by the rats on their posterior paws in comparison with the control group (*p <* 0.05 and *p <* 0.01). The lack of an antinociceptive response upon PEA acute administration was highlighted in the control animals (vehicle + vehicle). The efficacy of acute PEA to generate analgesia in Groups d and g was monitored daily until Day 36, finding it necessary to improve the daily dose of PEA up to 120 mg kg^−1^ to maintain a stable analgesic effect (*p <* 0.05) (Figure 3a,b).

### 3.2. PEA Reduces the Astrocyte Activation Evoked by Opioids

**Figure 4:**
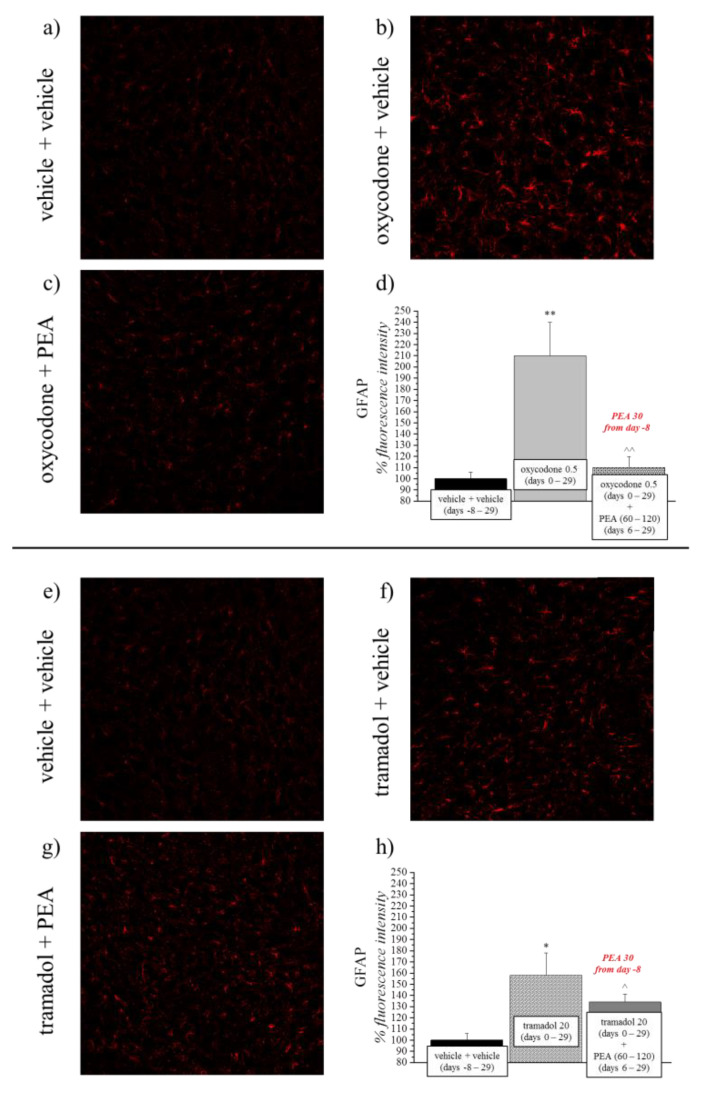
Effect of repeated administrations of PEA on the astrocytic profile in the lumbar spinal cord. Rats were treated with the vehicle or pre-emptive PEA (30 mg kg^−1^) p.o. daily (in the evening) for the duration of the experiment, starting on Day −8. (**a**–**d**) Oxycodone (0.5 mg kg^−1^ s.c.) was administered from Day 1 until the complete loss of the antinociceptive response was achieved (Day 29). From Day 6, animals treated with oxycodone + pre-emptive PEA received an additional acute daily PEA treatment (60–120 mg kg^−1^) in co-administration with the opioid until Day 29. (**e**–**h**) Tramadol (20 mg kg^−1^ s.c.; Group g) was injected from Day 1 until the complete loss of the antinociceptive response was achieved (Day 29). From Day 6, animals treated with tramadol + pre-emptive PEA received an additional acute daily PEA treatment (60–120 mg kg^−1^) in co-administration with the opioid until Day 29. The number of GFAP-positive cells was counted in the dorsal horn of the L4–L5 spinal cord. Transverse sections of spinal cord were imaged at ×20 objective (scale bar = 50 μm). Histograms show a quantitative analysis of the GFAP fluorescence intensity. Each value represents the mean ± S.E.M. of six rats, obtained by analysing four independent fields for both sides of the dorsal horn of the lumbar spinal cord. Statistical analysis was one-way ANOVA followed by Bonferroni’s post hoc comparison. * *p <* 0.05 and ** *p <* 0.01 vs. vehicle + vehicle group; ^ *p <* 0.05 and ^^ *p <* 0.01 vs. oxycodone or tramadol + vehicle treated animals.

At the end of the behavioural experiments and when tolerance to the antinociceptive effect of opioids was still in place (Day 29), the dorsal horn of the lumbar spinal cord was analysed in different groups of animals treated with different regimens: vehicle + vehicle, oxycodone + vehicle, oxycodone + PEA (Group c), tramadol + vehicle and tramadol + PEA (Group f) (Figure 4a,b).

Repeated oxycodone treatment triggered the activation of astrocytes, evaluated immunohistochemically with an anti-GFAP-antibody (*p <* 0.01). PEA avoided the maladaptive plasticity induced by the opioid, quantified as the percentage of GFAP fluorescence intensity (*p <* 0.01) (Figure 4a). Tramadol repeated injections also increased the GFAP fluorescence intensity in comparison with the control group (*p <* 0.05), indicating that the PEA treatment had rebalanced the astrocyte plasticity (*p <* 0.05) (Figure 4b).

### 3.3. Different Combinations of PEA with Tramadol or Oxycodone Modulate Opioids’ Analgesia

**Figure 5:**
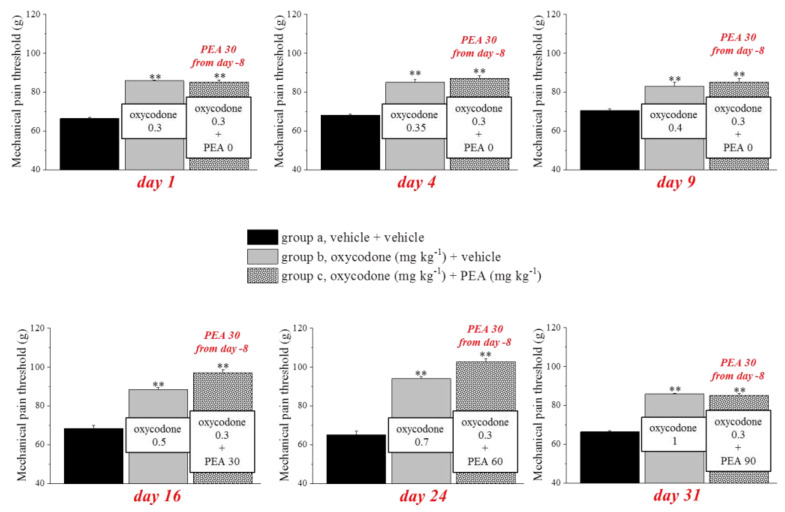
Antinociceptive effects of different combinations of oxycodone and PEA. Animals were treated with the vehicle (Groups a and b) or pre-emptive PEA (30 mg kg^−1^ in the evening; Group c) per os daily for the duration of the experiment, starting on Day −8. To maintain a pain threshold of about 90 ± 10 g, beginning on Day 1, increasing daily doses of oxycodone (0.3–1 mg kg^−1^) were injected s.c. to Group b. Different combinations of oxycodone (0.3 mg kg^−1^, s.c.) and PEA (30–90 mg kg^−1^, p.o.) were administered to Group c. Measurements were conducted every day in the morning, before and 30 min after oxycodone and/or acute PEA administration. Representative results obtained 30 min after treatments on Days 1, 4, 9, 16, 24 and 31 are shown. The results are expressed as mean ± SEM of values from 12 rats analysed in two different experimental sets. Statistical analysis was one-way ANOVA followed by Bonferroni’s post hoc comparison. ** *p <* 0.01 vs. vehicle + vehicle group.

**Figure 6:**
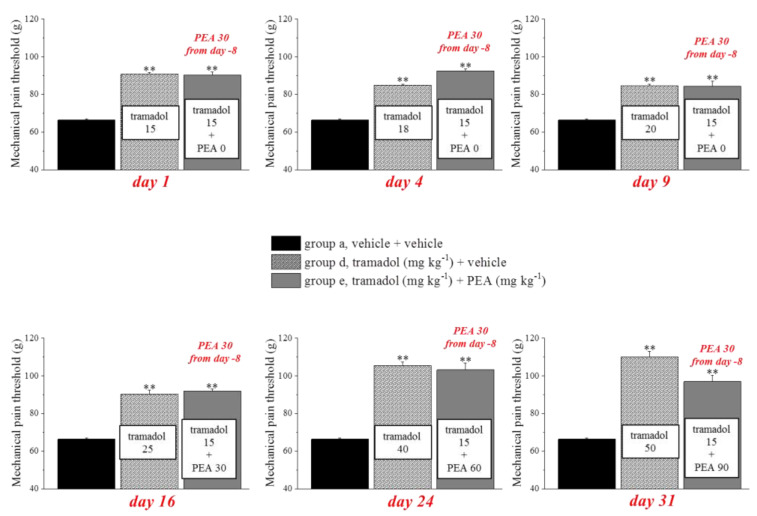
Antinociceptive effects of different combinations of tramadol and PEA. Rats were treated with the vehicle (Groups a and d) or pre-emptive PEA (30 mg kg^−1^ in the evening; Group e) per os daily for the duration of the experiment, starting on Day −8. To maintain a pain threshold of about 90 ± 10 g, beginning on Day 1, increasing daily doses of tramadol (15–50 mg kg^−1^) were injected s.c. into Group d. Different combinations of oxycodone (15 mg kg^−1^, s.c.) and PEA (30–90 mg kg^−1^, p.o.) were administered to Group e. Measurements were conducted every day in the morning, before and 30 min after tramadol and/or acute PEA administration. Representative results obtained 30 min after treatments on Days 1, 4, 9, 16, 24 and 31 are shown. The results are expressed as mean ± SEM of values from 12 rats analysed in two different experimental sets. Statistical analysis was one-way ANOVA followed by Bonferroni’s post hoc comparison. ** *p <* 0.01 vs. vehicle + vehicle group.

Previous experiments showed the capacity of PEA to modulate morphine’s antinociception [9]. In accordance with this evidence, we set up a new experiment focused on evoking stable analgesia based on the use of pre-emptive PEA followed by the co-administration of increasing doses of acute PEA with oxycodone or tramadol. For both opioids, the purpose was to reach daily, over time, the same increase in the pain threshold (arbitrarily fixed at 90 ± 10 g, starting from a basal value of about 65 g) by using the lowest doses of opioids in association with PEA. Animals were administered the vehicle or pre-emptive PEA (30 mg kg^−1^) per os daily (in the evening) from Day −8 until the end of the experiments. From Day 1 to Day 31, we measured the pain threshold daily before and 30 min after acute treatment with increasing doses of oxycodone (0.3–1 mg kg^−1^, s.c.) or different combinations of oxycodone (0.3 mg kg^−1^, s.c.) and PEA (30–90 mg kg^−1^, p.o.; Figure 5), and increasing doses of tramadol (15–50 mg kg^−1^, s.c.) or different combinations of tramadol (15 mg kg^−1^, s.c.) and PEA (30–90 mg kg^−1^, p.o.; Figure 6). Analgesia was measured using a mechanical noxious stimulus (paw pressure test). Figure 5 and Figure 6 show the results of representative days obtained during these studies. On Day 1, 0.3 mg kg^−1^ oxycodone was necessary to increase the pain threshold up to 90 g in both groups (*p <* 0.01). On Day 4, 0.35 mg kg^−1^ oxycodone was needed to reach the same antinociceptive effect evoked on Day 1 (*p <* 0.01) whereas 0.3 mg kg^−1^ was enough for the group of animals treated with pre-emptive PEA (*p <* 0.01). On Day 9, 0.4 mg kg^−1^ was used in the oxycodone + vehicle group to maintain stable analgesia to around 90 g, while 0.3 mg kg^−1^ was necessary in oxycodone + PEA-treated animals (*p <* 0.01) (Figure 5). Oxycodone at 0.5 mg kg^−1^ was active on Day 16 in the oxycodone + vehicle group (*p <* 0.0) and only 0.3 mg kg^−1^ was necessary when combined with 30 mg kg^−1^ PEA in oxycodone + PEA-treated animals (*p <* 0.0). On the last day of treatment (Day 31), we used 1 mg kg^−1^ oxycodone compared with 0.3 mg kg^−1^ oxycodone in addition to 90 mg kg^−1^ PEA in order to obtain the same analgesia.

The protocol adopted for the study involving tramadol made it necessary to use 15 mg kg^−1^ of the opioid in both groups (tramadol + vehicle and tramadol + PEA) on Day 1 to increase the animal’s pain threshold up to 90 g (*p <* 0.01). On Day 4, 18 mg kg^−1^ of tramadol was necessary in the tramadol + vehicle group (*p <* 0.01) in comparison with 15 mg kg^−1^ used in the tramadol + PEA group (*p <* 0.01). On Day 16, to maintain the analgesic effect at 90 g, we were forced to injected 25 mg kg^−1^ tramadol (*p <* 0.01) (tramadol + vehicle) in comparison with 15 mg kg^−1^ of the opioid in combination with 30 mg kg^−1^ PEA (*p <* 0.01). On the last day, we used 50 mg kg^−1^ tramadol, and 15 mg kg^−1^ tramadol combined with 90 mg kg^−1^ PEA (Figure 6).

**Figure 7:**
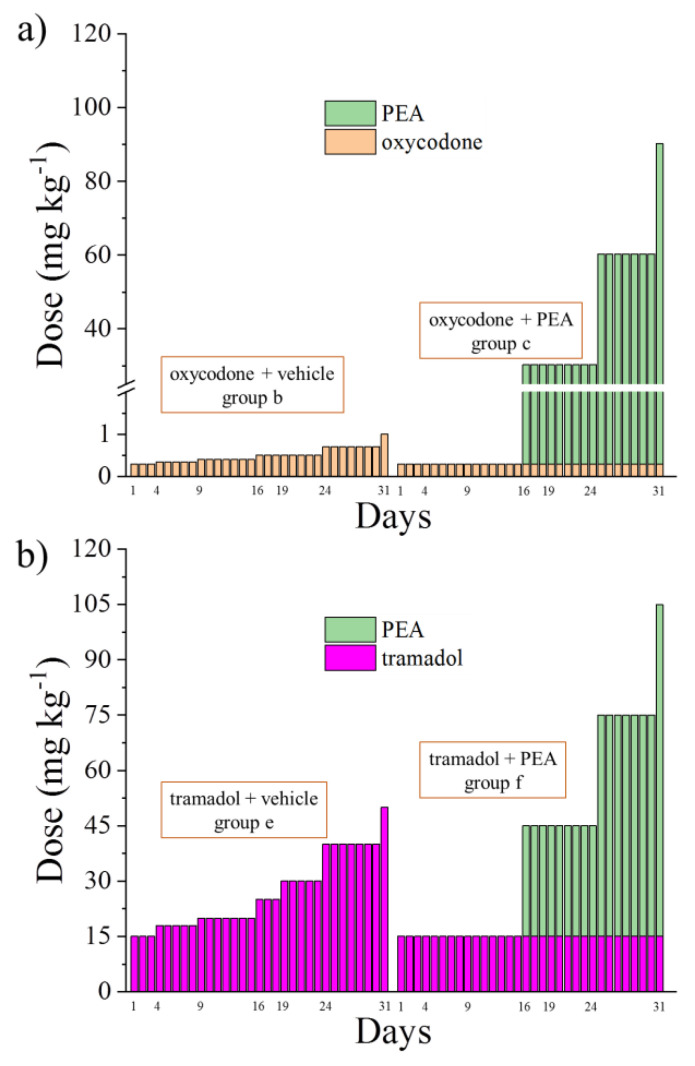
Comparison among equally analgesic dosages of oxycodone–PEA or tramadol–PEA combinations, and oxycodone or tramadol alone. Animals were treated with the vehicle or pre-emptive PEA (30 mg kg^−1^) p.o. daily (in the evening) for the duration of the experiment, starting on Day −8. To maintain a significant daily increase in the pain threshold (90 ± 10 g) vs. the baseline, from Day 1 to Day 31, increasing doses of (**a**) oxycodone (0.3–1 mg kg^−1^) or (**b**) tramadol (15–50 mg kg^−1^) were injected s.c. Different combinations of (**a**) oxycodone (0.3–1 mg kg^−1^) or (**b**) tramadol (15–50 mg kg^−1^) and PEA (30–90 mg kg^−1^, p.o.) were administered to Groups c and f, respectively. The dosages (in mg kg^−1^) necessary every day to maintain the required antinociceptive effect evaluated by the paw pressure test (Days 1–31) are reported. Measurements were performed in the morning, 30 min after opioids or/and acute PEA administration. Each group consisted of 12 rats analysed in two different experimental sets.

In Figure 7, the trend of an increasing dosage from Day 1 to Day 31 is summarized for both experiments. Figure 7a shows the doses of oxycodone (orange columns) and PEA (green columns) used in the oxycodone + vehicle group vs. oxycodone + PEA group over time to reach a stable antinociception of about 90 g. Figure 7b summarizes the doses of tramadol (purple columns) and PEA (green columns) used over time in the tramadol + vehicle group vs. the tramadol + PEA group.

### 3.4. Effect of PEA Treatment on Ex Vivo Analysis

As described for the previous experiments, at the end of the behavioural evaluations the animals were sacrificed to perform the ex vivo analysis.

**Figure 8:**
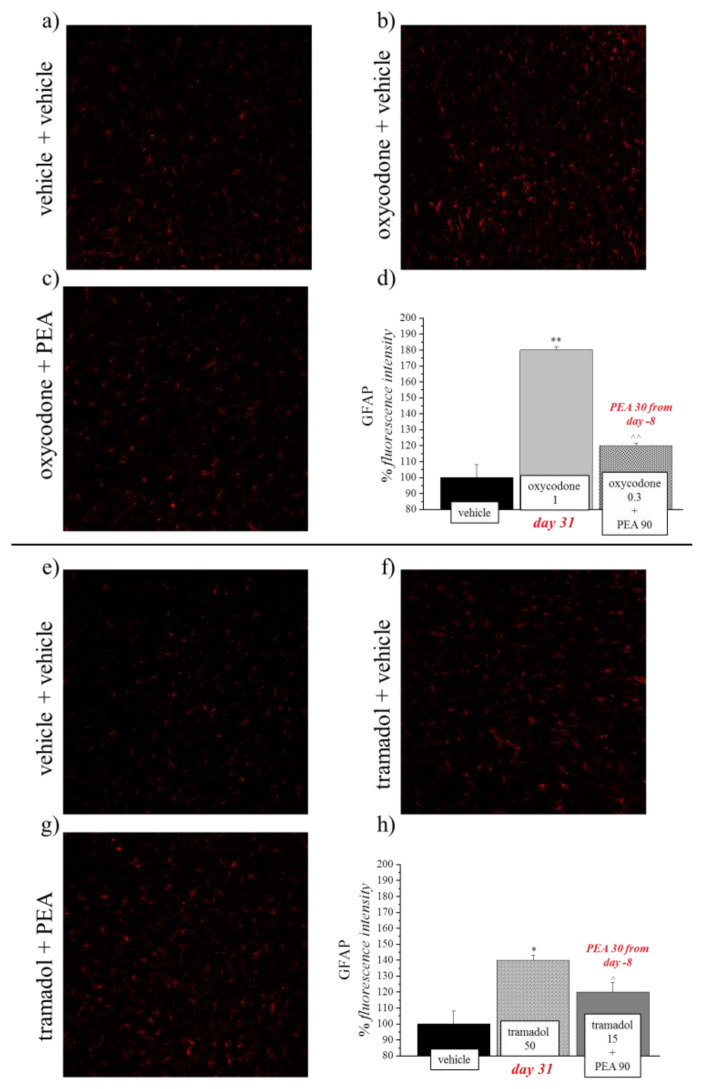
Effect of different combinations of opioids and PEA on the astrocytic profile in the lumbar spinal cord. Rats were treated with the vehicle or pre-emptive PEA (30 mg kg^−1^) p.o. daily (in the evening) for the duration of the experiment, starting on Day −8. To maintain a significant increase in the pain threshold (90 ± 10 g) vs. baseline, beginning on Day 1, daily increasing doses of (**b**) oxycodone (0.3–1 mg kg^−1^) or (**f**) tramadol (15–50 mg kg^−1^) were injected s.c. Different combinations of (**c**) oxycodone (0.3 mg kg^−1^) or (**g**) tramadol (15 mg kg^−1^) and PEA (30–90 mg kg^−1^, p.o.) were administered to Groups c and f, respectively. Control animals (**a**,**e**) were treated with vehicles. On Day 31, the number of GFAP-positive cells was measured in the dorsal horn of the L4–L5 spinal cord. Transverse sections of the spinal cord imaged at ×20 objective (scale bar = 50 μm. Histograms (**d**,**h**) show a quantitative analysis of the GFAP fluorescence intensity. Each value represents the mean ± S.E.M. of six rats, obtained by analysing four independent fields for both sides of the dorsal horn of the lumbar spinal cord. Statistical analysis was one-way ANOVA followed by Bonferroni’s post hoc comparison. * *p <* 0.05 and ** *p <* 0.01 vs. vehicle + vehicle group; ^ *p <* 0.05 and ^^ *p <* 0.01 vs. oxycodone or tramadol + vehicle treated animals.

Increasing doses of oxycodone (0.3–1 mg kg^−1^) triggered astrocyte activation in the dorsal horn of the lumbar spinal cord evaluated immunohistochemically with an anti-GFAP-antibody (*p <* 0.01) (Figure 8d) with respect to the control group (vehicle + vehicle). PEA significantly reduced the percentage of GFAP fluorescence intensity in comparison with the oxycodone + vehicle group, reducing the maladaptive plasticity evoked by the opioid (*p <* 0.0). Tramadol also significantly increased the percentage of GFAP fluorescence intensity (*p <* 0.05) in comparison with the control group, which was reduced by PEA treatment (*p <* 0.05) (Figure 8h).

**Figure 9:**
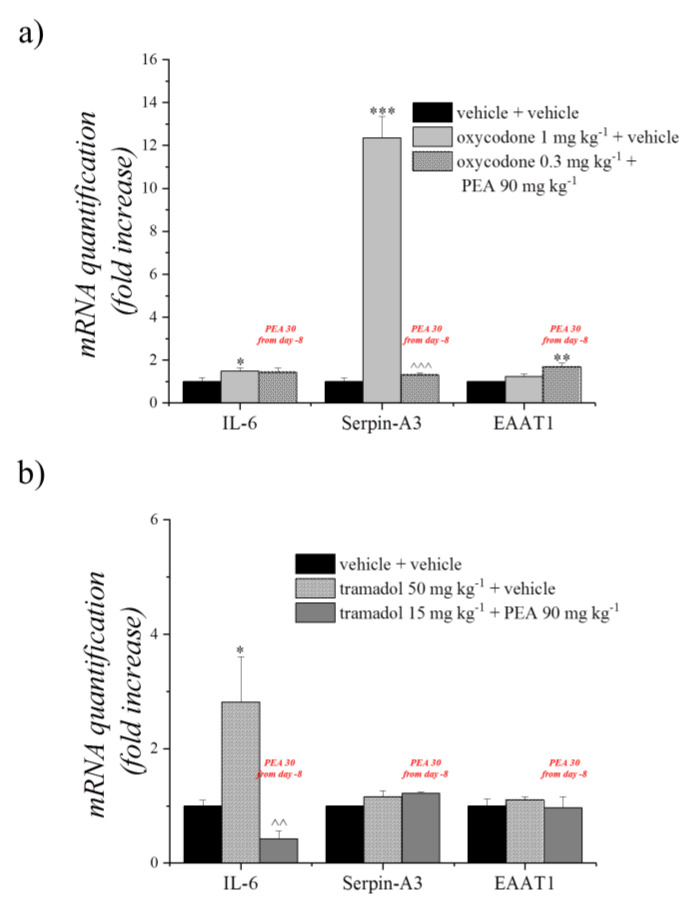
Effect of different combination of opioids and PEA on mRNA expression profiles in the lumbar spinal cord. Animals were orally treated with the vehicle or pre-emptive PEA (30 mg kg^−1^ daily in the evening) for the duration of the experiment, starting on Day −8. To maintain a significant increase in the pain threshold (90 ± 10 g) vs. the baseline, beginning on Day 1, daily increasing doses of (**a**) oxycodone (0.3–1 mg kg^−1^), or oxycodone (0.3 mg kg^−1^) and PEA (30–90 mg kg^−1^, p.o.) or (**b**) tramadol (15–50 mg kg^−1^), or tramadol (15 mg kg^−1^) and PEA (30–90 mg kg^−1^, p.o.) were injected s.c. On Day 31, the expression levels of IL-6, Serpin-A3 and EAAT1 genes were measured in the spinal cord. Each value represents the mean ± S.E.M. of three rats. Statistical analysis was one-way ANOVA followed by Bonferroni’s post hoc comparison. * *p <* 0.05, ** *p <* 0.01 and *** *p <* 0.001 vs. vehicle + vehicle group; ^^ *p <* 0.01 and ^^^ *p <* 0.001 vs. oxycodone or tramadol + vehicle treated animals.

The RT-PCR analysis performed on the spinal cord of rats at Day 31 revealed a significant increase in IL-6 expression in the oxycodone + vehicle (*p <* 0.05) and tramadol + vehicle (*p <* 0.05) groups (1.49 ± 0.15 and 2.81 ± 0.8 respectively) vs. the control group. PEA treatment was able to counteract the increased expression in tramadol-treated rats (*p <* 0.01) (tramadol + PEA group; 0.42 ± 0.14). No significant effect was exerted by PEA on oxycodone-treated animals. Serpin-A3 was highly upregulated in the oxycodone + vehicle group (*p <* 0.001) (12.36 ± 0.99) and PEA strongly reduced its expression (*p <* 0.001) (1.31 ± 0.09). EAAT1 was overexpressed in the oxycodone + PEA group (12.36 ± 0.99) vs. the control group (*p <* 0.01) (Figure 9a), while no statistical differences were observed in the expression of Serpin-A3 and EAAT1 genes in tramadol-treated rats (Figure 9b).

**Figure 10:**
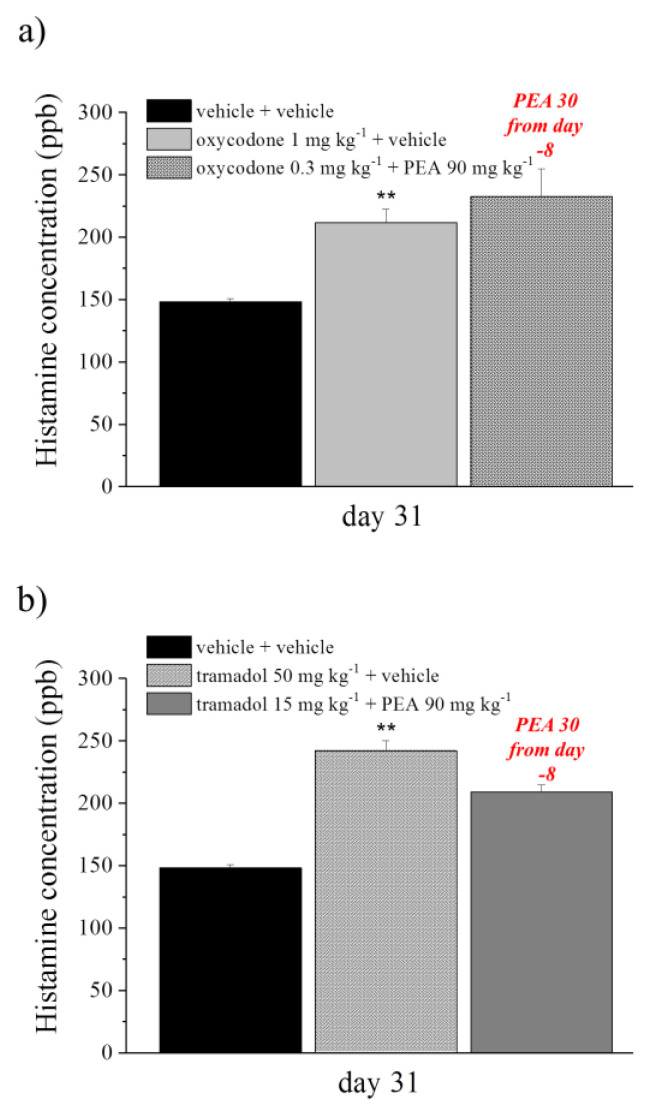
Effect of different combination of opioids and PEA on histamine plasma levels. Animals were orally treated with the vehicle or pre-emptive PEA (30 mg kg^−1^ daily in the evening) for the duration of the experiment, starting on Day −8. To maintain a significant increase in the pain threshold (90 ± 10 g) vs. the baseline, beginning on Day 1, increasing daily doses of (**a**) oxycodone (0.3–1 mg kg^−1^), or oxycodone (0.3 mg kg^−1^) and PEA (30–90 mg kg^−1^, p.o.) or (**b**) tramadol (15–50 mg kg^−1^), or tramadol (15 mg kg^−1^) and PEA (30–90 mg kg^−1^, p.o.) were injected s.c. On Day 31, the histamine concentration was measured in the plasma by HPLC analysis. Data represent the mean ± S.E.M. of 12 rats. Statistical analysis was one-way ANOVA followed by Bonferroni’s post hoc comparison. ** *p <* 0.01 vs. vehicle + vehicle group.

The repeated opioid treatments determined a significant upregulation of histamine plasma levels, as shown in Figure 10 (*p <* 0.01). No statistically significant effect was exerted by PEA.

## 4. Discussion

In the present study, we demonstrated that ultramicronized PEA behaves as an adjuvant analgesic relative to tramadol and oxycodone, by limiting tolerance and exerting a dose-sparing effect. Similar findings have been recently reported by our group through combining PEA with morphine [9]. In particular, pre-emptive administration of ultramicronized PEA significantly delayed the development of tramadol (but not oxycodone) tolerance, the findings being in line with previous results on PEA’s ability to delay the onset of morphine tolerance, doubling the days for which naïve rats are responsive to morphine [8]. Moreover, all the effects cited above matched a reduction in astrocyte activation in the lumbar spinal cord of rats treated with the opioids in combination with PEA.

Pain can be classified as physiological (acute) or pathological (persistent). As a mechanism-based classification, ‘physiological pain’ refers to pain attributable to the activation of the peripheral receptive terminals of primary afferent neurons in response to noxious chemical, mechanical or thermal stimuli [27]. It acts as an important alarm system, alerting us to external danger or internal problems, and it continues only as long as the noxious stimulus is maintained [28]. In contrast, neuropathic pain, as an example of pathological pain, usually results from damage to the neural tissue by a disease, toxin or infection [29]. Given the prevalence of persistent pain in adults and the disabling effects it entails, opioids are still considered the best painkillers. However, the benefits of opioids prescribed for a long-term treatment can result in the appearance of common unwanted side effects such as constipation, nausea and vomiting, and in more severe side effects such as addiction, physical dependence and respiratory depression [30] when escalating from appropriate opioid use to misuse [31]. Moreover, motor behaviour alterations evoked by repeated morphine injections were also recently highlighted in rats [32].

Clinically speaking, another limit to their chronic use in practice is the development of tolerance, which is illustrated by a reduced responsiveness to an opioid agonist, and is manifested by the need to use increasing doses to reach the desired effect until reaching a dose that is no longer therapeutic but toxic. Indeed, more than 10-fold dose escalations of opioid dose in chronic pain management are common [33].

In a recent work, it emerged that the morphine starting dose, the dosing frequency, the increments and the timing are responsible for the onset of antinociceptive tolerance [34]. Additionally, the complexity of chronic pain syndromes requires tailored pharmacological interventions and innovative drugs to effectively and safety control pain. Some studies have reported how different experimental drugs can be administered in combination (if they prove to be safe) with clinical opioids (e.g., intrathecal ziconotide, an *N*-type calcium ion channel inhibitor, ω-conotoxins with intrathecal morphine [35] or metformin, a potent antidiabetic drug [36]) to provide better pain relief and fewer adverse events than opioids alone. Moreover, the management of ongoing pain has been improved by the use of long-acting opioid agonists, and new formulations have upgraded the management of breakthrough pain [37]; however, efforts to overcome the adverse effects of opioids have still met with limited success.

PEA is an endogenous lipid compound, i.e., the amide of ethanolamide and palmitic acid. It was first isolated from soy lecithin and was shown to be produced ‘on demand’ by mammals in response to stressful conditions [38]. The first observation in this regard came from the infarcted canine myocardium [39]. Since then, several studies have characterized the changes in PEA’s metabolic pathways and tissue levels in different pathological conditions [40,41,42,43], with decreased levels sustaining disease development [12,44,45]. According to one of the most widely accepted hypotheses, PEA levels increase when tissues are faced with an actual or potential injury, and such an increase serves as an early warning signal to counteract injury and inflammation [46]. In view of this, it could be argued that pathological conditions may arise in which endogenous levels of PEA are inadequate for dealing with the ensuing insult. In these cases, a viable approach may be the exogenous administration of PEA to effectively ‘recharge’ the body’s own supply, provided that PEA is supplied in bioavailable forms [47]. This is the case for ultramicronized PEA, whose plasma levels following oral administration are fivefold higher compared with naïve PEA [21].

As mentioned before, our previous studies demonstrated the capacity of ultramicronized PEA to delay the onset of morphine tolerance, doubling the days in which naïve rats are responsive to the morphine antinociceptive effect [8]. Moreover, newly adopted protocols highlighted that a pre-emptive PEA treatment was able to potentiate morphine analgesia, making it possible for an opioid dose reduction to maintain stable analgesia over time when the opioid was co-administered with the amide [9]. This study extended the previous findings regarding the efficacy of PEA in modulating opioids’ analgesia to other two analgesics: tramadol and oxycodone. Oxycodone is a strong m-opioid agonist, quite similar in analgesic potency to morphine [48], and it is used orally for cancer pain [49] and parentally for post-operative pain [50,51]. Tramadol is a centrally acting analgesic drug with a low affinity for m-opioid receptors. It has been shown to be about 1/6–1/10 as potent an analgesic as morphine, considering both the intensity and duration of the effects [52]. In the first protocol adopted here, we showed that pre-emptive treatment with ultramicronized PEA (30 mg kg^−1^ PEA from Day −8 until the end of the experiment) was able to prolong oxycodone analgesia. Interestingly, we also highlighted how a new daily treatment with PEA, in co-administration with the opioids, was capable to enhance oxycodone and tramadol analgesia in comparison with the group of animals managed only with pre-emptive PEA. As verified by our previous work, the extension of opioid analgesia can be achieved in the absence of pre-treatment with PEA [8], while it is necessary to boost morphine’s efficacy [9]. Moreover, we previously found that recovery from morphine tolerance was speeded up in animals treated with PEA, suggesting that this amide can promote the phenomenon of cellular plasticity that is able to antagonize the complex mechanisms involved in the development of tolerance [9].

As reported in the literature, PEA is unable to modify the normal pain threshold of naïve animals [24,53] but is active in conditions of hypersensitivity generated by trauma [13,54] or chemotherapy treatments [24]. It is important to emphasize that the tolerance phenomenon shares several similarities with the alterations evoked by a neuropathic pain condition; among these, we include changes affecting the glial cells, since there is evidence that specific inhibitors of the microglia and astrocytes are able to inhibit both processes [10,26].

Since PEA is a modulator of the glial response [7], we tested its effect in tolerant animals. When oxycodone and tramadol tolerance was well established (from Day 31), PEA exerted antinociceptive effects upon its acute administration in rats previously pre-treated with PEA. These results are in accordance with our previous work, in which PEA determined analgesia in morphine-tolerant animals pre-treated with the amide and lacked efficacy in morphine-tolerant rats pre-treated with only the vehicle [9]. This evidence led us to set up a protocol in which we maintained stable antinociception based on the use of pre-emptive PEA followed by the acute administration of PEA with tramadol or oxycodone. Clinically speaking, this could be reasonably considered an outstanding finding, since one of the main limits of chronic opioid use is the need to increase the dose needed to achieve the desired effect until the dose may become toxic.

The results obtained matched with the modulatory role of PEA in the nervous system. PEA is thought to be the first ALIAmide and is also the most studied so far. Its anti-inflammatory and immuno-modulating properties were defined in 1957 after its isolation from egg yolk [55,56]. However, it was with the work of the Nobel prize winner Rita Levi-Montalcini that PEA regained attention in the 1990s, revealing its antinociceptive and powerful anti-inflammatory effects [57].

Three mechanisms have been proposed so far to explain PEA’s properties. The first supposes that PEA acts via an ALIA mechanism; hence the name of this class of substances (ALIAmides) that share the ability to settle the excess reactivity of non-neuronal and mast cells by downregulating the degranulation of the latter [58,59]. The second mechanism supposes that PEA acts throughout the activation of the nuclear peroxisome proliferator-activated receptor-a (PPAR-α) [60] and the orphan receptor G-protein coupling (GPR55). In particular, PPAR-α its important because belongs to a group of nuclear receptor proteins that normalize the expression of genes involved in pro-inflammatory processes [61]. The last mechanism proposes an ‘entourage effect’, which hypothesizes an indirect effect of PEA as a substance that enhances the anti-inflammatory effects of other molecules [62]. Focusing on the inflammatory markers, we highlighted how PEA determined a reduction in IL-6 and serpin-A3 mRNA levels in the oxycodone- and tramadol-treated groups, respectively. In particular, it has emerged that patients with higher serum IL-6 have worse tramadol tolerance. Indeed, Tanaka and colleagues demonstrated that the early discontinuation of oral tramadol affected most of the patients with higher IL-6 levels in the serum who switched to strong opioid analgesics without dose escalation caused by inadequate pain relief [63]. Regarding the data about Serpin-A3 levels, to the best of our knowledge, this is the first evidence of its increase in oxycodone-tolerant-rats.

Chronic exposure to morphine induced activation of glial cells, which contributes negatively to the analgesic response of the opioid [8,64]. Activated by stressors, parenchymal microglia and astrocytes lead to the formation of and the subsequent production of cytokines, chemokines, cellular adhesion molecules and surface antigens to strengthen the immune cascade in the central nervous system. The pro-inflammatory cytokines (IL-1 β, IL-6 and TNF) released by the glial cells were observed after the induction of morphine tolerance [65]. To validate the role of neuroinflammation in morphine tolerance, it has been demonstrated that this phenomenon can be inhibited by the application of proinflammation cytokine antagonists, such as the soluble tumour necrosis factor (TNF) receptor, anti-IL-6 antibodies and the IL-1 receptor antagonist [66]. Moreover, repeated morphine injections increased microglia and astrocyte cell density [8], and evoked a morphological arrangement in astrocytes characterized by an increase in the number and length of total and secondary processes [67]. As already mentioned, the administration of glial inhibitors counteracts the phenomenon of tolerance [10,11], and PEA seems to act in a similar way as a modulator of glial cells. In this study, we confirmed the role of astrocytes in the development of opioid tolerance and for the progressive reduction in their antinociceptive effects. To the best of our knowledge, this is the first evidence that highlights astrocytes activation in the dorsal horn of the spinal cord of naïve animals caused by repeated tramadol and oxycodone treatments. In this context, PEA was shown to have inhibitory properties to counteract the maladaptive plasticity that occurs in astrocytes during repeated treatment with tramadol and oxycodone. One explanation for the reduction in the side effects of the opioids could be through a glial-mediating mechanism.

Although glial cell activation is widely accepted as an important contribution to the pathophysiology of opioids’ side effects, as previously described, we have to remember that glial cells also respond to the inflammatory stimuli produced by other immune cells, demonstrating that there is a crosstalk between the CNS and the immune system [47,68]. In this perspective, mast cells are an important link between the two systems that PEA can modulate. Mast cells are the first to intervene during an insult, and to amplify and prolong the immune and nerve responses that arise from their activation [61]. Moreover, mast cell degranulation provides a vast number of mediators such as cytokines, histamine and soluble factors that sensitize nociceptors, contributing to pain chronicization [69,70]. The study highlighted how histamine increased in the plasma of tolerant animals even if PEA did not induce a significant reduction, suggesting a more complex mechanism of action that involves not only histamine but also a plethora of other mediators controlled by mast cells.

## 5. Conclusions

In conclusion, the present study provides clear evidence that supplementation with ultramicronized PEA can be a valid strategy to reduce tramadol and oxycodone dose escalation during a long-term treatment and to delay the onset of opioid tolerance. This effect allows us to maintain a stable and long-lasting analgesic effect that, clinically speaking, is crucial during drug therapy for the management of persistent pain.

## Data Availability

The data presented in this study are available on request from the corresponding author.
